# Association Between Triglyceride Glucose Index and Non-Small Cell Lung Cancer Risk in Chinese Population

**DOI:** 10.3389/fonc.2021.585388

**Published:** 2021-03-11

**Authors:** Xin Yan, Yujuan Gao, Jingzhi Tong, Mi Tian, Jinghong Dai, Yi Zhuang

**Affiliations:** Department of Respiratory and Critical Care Medicine, Nanjing Drum Tower Hospital, The Affiliated Hospital of Nanjing University Medical School, Nanjing, China

**Keywords:** triglyceride glucose index, non-small cell lung cancer, insulin resistance, predictor, homeostasis model assessment of insulin resistance

## Abstract

**Background:**

Numerous studies showed that insulin resistance (IR) was associated with cancer risk. However, few studies investigated the relationship between IR and non-small cell lung cancer (NSCLC). The aim of this study is to explore the association of triglyceride glucose (TyG) index, a simple surrogate marker of IR, with NSCLC risk.

**Methods:**

791 histologically confirmed NSCLC cases and 787 controls were enrolled in the present study. Fasting blood glucose and triglyceride were measured. The TyG index was calculated as ln [fasting triglycerides (mg/dl) ×fasting glucose (mg/dl)/2]. Logistic regression analysis was performed to estimate the relationship between NSCLC risk and the TyG index.

**Results:**

The TyG index was significantly higher in patients with NSCLC than that in controls (8.42 ± 0.55 *vs* 8.00 ± 0.45, *P* < 0.01). Logistic regression analysis showed that the TyG index (*OR* = 3.651, 95%*CI* 2.461–5.417, *P* < 0.001) was independently associated with NSCLC risk after adjusting for conventional risk factors. In addition, a continuous rise in the incidence of NSCLC was observed along the tertiles of the TyG index (29.4 *vs* 53.8 *vs* 67.2%, *P* < 0.001). However, there were no differences of the TyG index in different pathological or TNM stages. In receiver operating characteristic (ROC) curve analysis, the optimal cut-off level for the TyG index to predict incident NSCLC was 8.18, and the area under the ROC curve (AUROC) was 0.713(95% *CI* 0.688–0.738).

**Conclusions:**

The TyG index is significantly correlated with NSCLC risk, and it may be suitable as a predictor for NSCLC.

## Introduction

The 2018 Global Cancer Report (GLOBOCAN) showed that lung cancer is the most commonly diagnosed cancer (11.6% of the total cases) and the leading cause of cancer death (18.4% of the total cancer deaths) (1). The incidence of lung cancer in China in 2015 was 69% in males and 31% in females; it is still increasing over recent years. Lung cancer contributes to 30% of all cancer deaths in China (2, 3), which represents a huge clinical burden and public health attention. Non-small cell lung cancer (NSCLC) is the most common subtype of lung cancer and accounts for almost 80% of all cases. Smoking has been identified as a major risk factor for lung cancer (4, 5), but the incidence of lung cancer in non-smokers has also increased. Therefore, there is an urgent need to study other modifiable risk factors for lung cancer.

Insulin resistance (IR) plays a key role in the pathophysiology of type 2 diabetes (T2DM). In addition, IR is a sign of obesity, metabolic syndrome (MetS), and non-alcoholic fatty liver disease (NAFLD) (6, 7). Hyperinsulinemia is characteristic of insulin resistance and may promote cell proliferation (8). Previous studies have evaluated the relationship between insulin resistance and the development of cancers such as prostate cancer and thyroid cancer (9, 10). However, studies remain conflicting regarding the relationship between lung cancer and insulin resistance, as positive or invalid association has been reported (11, 12). Assessment of the homeostasis model of insulin resistance based on fasting glucose and insulin levels (HOMA-IR) is a widely used surrogate indicator of IR in clinical practice (13). However, plasma insulin levels are usually measured in diabetic patients, which are not suitable for the general population. Recently, the triglyceride glucose (TyG) index calculated from fasting triglyceride (TG) and glucose levels has become a reliable alternative indicator of IR (14). In predicting some diseases including non-alcoholic fatty liver disease (NAFLD) and arterial stiffness, the TyG index is even superior to HOMA-IR (15, 16). However, few studies have evaluated the TyG index in the context of NSCLC. The present study aims to elucidate the potential relationship between NSCLC and the TyG index.

## Participants and Methods

### Study Population

We retrospectively collected 791 newly diagnosed and pathologically confirmed NSCLC patients between 2016 and 2018 at the Department of Respiration of Nanjing Drum Tower Hospital. 787 healthy adults without NSCLC were randomly selected following examination in the clinic and were classified as the control group. All subjects gave informed written consent to participate. Participants with previous history of cancer, diabetes, history of usage of fenofibrate triglyceride-lowering drugs, and liver, kidney or other diseases associated with lipid metabolism disorders were excluded. The study was approved by the Human Research Ethics Committee of Nanjing Drum Tower Hospital.

### Physical Examination and Biochemical Tests

All participants’ weights and heights were measured using standardized stadiometers and scales while wearing light clothing without shoes. A standard questionnaire was used to evaluate smoking habit, history of acute and chronic illnesses, and drug use. Morning fasting venous blood samples from all participants were obtained and used to measure routine biochemical indexes. Fasting blood samples were collected after at least 10 h overnight and analyzed for the biochemical measurements. Fasting blood glucose (FPG), lipid profile including total cholesterol (TC), triglyceride (TG), high-density lipoprotein cholesterol (HDL-C) and low-density lipoprotein cholesterol (LDL-C), uric acid, and serum lactate dehydrogenase (LDH) were measured with commercial kits using an automated chemistry analyzer (Chemistry Analyzer Au2700, Olympus Medical Engineering Company, Japan). Blood routine examinations including white blood cell count (WBCC) and neutrophil count were determined using an automated blood cell counter (Beckman Coulter Ireland Inc. Mervue, Galway, Ireland).

### Statistical Analysis

Continuous variables were described by the mean (standard deviation) and compared by Student t-test or one-way ANOVA. Categorical variables were described by percentages (numbers) and compared by Chi-square test. The binary logistic regression analysis was performed to investigate the relationship between NSCLC risk and the TyG index after adjusting for potential confounders, including age and smoking status. Receiver operator characteristic (ROC) analyses were performed to calculate area under the ROC curve (AUROC) of TyG index for the incident of NSCLC. Data were analyzed using SPSS18.0 statistical software, with significance defined as *P <*0.05 (two-sided).

## Results

### Baseline Characteristics of the Study Population

Demographic and clinical characteristics of all participants were listed in **Table 1**. We identified 787 participants without lung cancer and 791 patients with newly diagnosed NSCLC. There were significant differences in age, sex ratio, smoking status between the two groups (all *P <*0.01). Hypertension was seen more frequently in the NSCLC group (*P* < 0.01). Moreover, patients with NSCLC had higher BMI, white blood cell count (WBCC), neutrophil count, TG, and TyG index than the control subjects (all *P* < 0.01). However, TC, HDL-C, and LDL-C levels were significantly lower in patients with NSCLC than those without lung cancer (all *P* < 0.01). The FPG level was similar between the two groups (*P* = 0.77).

**Table 1:** Demographic and clinical characteristics of all participants.

	Controls (n = 787)	NSCLC (n = 791)	P value
Age (years)	59.93 ± 10.73	61.75 ± 10.68	<0.01
Sex (Male/Female)	266/521	412/379	<0.01
Smoking (%)	92(11.68)	180(22.75)	<0.01
Hypertension (%)	67 (8.51)	257 (32.49)	<0.01
BMI (kg/m2)	22.96 ± 3.09	23.61 ± 3.05	<0.01
WBCC (×10^9/L)	5.46 ± 1.39	6.01 ± 2.02	<0.01
Neutrophil count (×10^9/L)	3.23 ± 1.10	3.67 ± 1.74	<0.01
FBG (mmol/L)	5.06 ± 0.63	5.08 ± 1.35	0.77
TC (mmol/L)	4.94 ± 0.98	4.15 ± 1.67	<0.01
TG (mmol/L)	0.82 ± 0.31	1.32 ± 0.87	<0.01
LDL-C(mmol/L)	3.01 ± 0.74	2.37 ± 0.71	<0.01
HDL-C(mmol/L)	1.50 ± 0.34	1.09 ± 0.31	<0.01
TyG index	8.00 ± 0.45	8.42 ± 0.55	<0.01
Uric acid (umol/l)	290.01 ± 75.48	328.81 ± 106.04	<0.01

Values are presented as mean ± standard deviation.

BMI, body mass index; WBCC, white blood cell counts; FBG, fasting blood glucose; TC, total cholesterol; TG, triacylglyceride; LDL-C, low-density lipoprotein cholesterol; HDL-C, high-density lipoprotein cholesterol; TyG, triglyceride and glucose index.

### The Relationship Between NSCLC Risk and the TyG Index by Binary Logistic Regression Analysis

The results of univariate logistic regression analysis indicated that a significant association of a high level of TyG index with NSCLC risk (*OR* = 5.883, 95%*CI* 4.616–7.498, *P*<0.001). In the multivariable logistic regression analysis, the correlation between the TyG index and NSCLC risk was also significant (*OR* = 3.651, 95%*CI* 2.461–5.417, *P* < 0.001) after adjusting for age, sex, smoking, BMI, hypertension, WBCC, Neutrophil count, TC, LDL-C, HDL-C, and uric acid (**Table 2**).

**Table 2:** Logistic regression analysis of TyG index and NSCLC risk.

Model	OR	95%CI	P
1	5.883	4.616–7.498	<0.001
2	4.821	3.728–6.234	<0.001
3	3.651	2.461–5.417	<0.001

Model1: unadjusted.

Model2: adjustment for age, sex, smoking, BMI, hypertension, WBCC, Neutrophil count.

Model3: adjustment for age, sex, smoking, BMI, hypertension, WBCC, Neutrophil count, TC, LDL-C, HDL-C and uric acid.

### The Incidence of NSCLC Compared Across the Tertiles of the TyG Index

**Figure 1:**
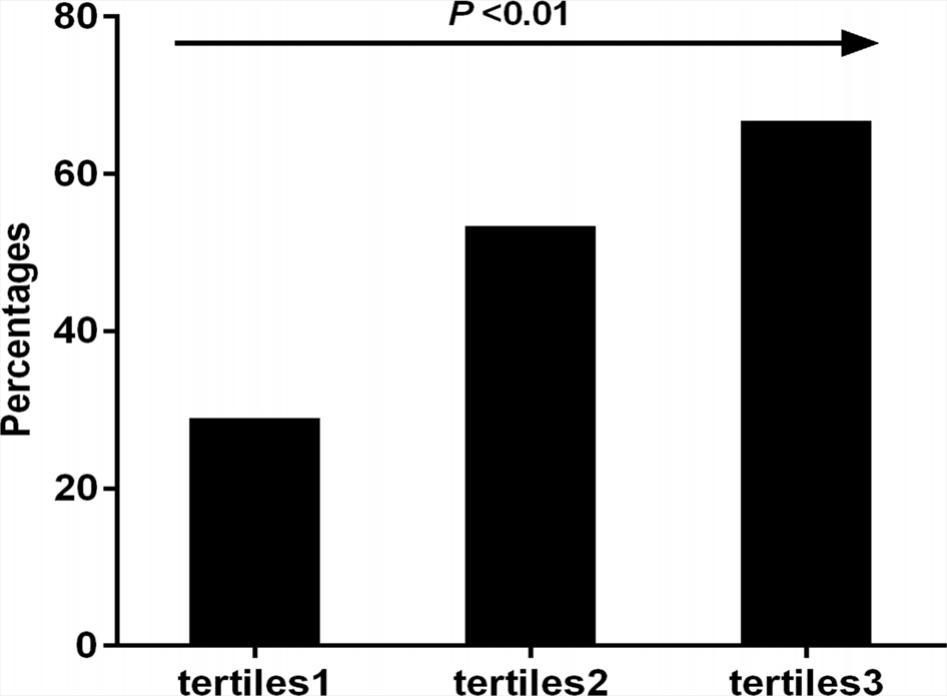
The incidence of NSCLC compared across the tertiles of the TyG index.

All participants were stratified into three groups based on the tertiles of their TyG index levels. **Figure 1** showed that a continuous rise in the incidence of NSCLC was observed along the tertiles of the TyG index (29.4 *vs* 53.8 *vs* 67.2%, *P* < 0.001).

### The TyG Index in Different Histopathological Type or TNM Stage

Patients with NSCLC were divided in to three groups according to histopathological classification: adenocarcinoma (n = 661), squamous cell carcinoma (n = 107) and other types (n = 23). Although patients with adenocarcinoma had higher TyG index than those with squamous cell carcinoma and other types (8.44 ± 0.56 *vs* 8.37 ± 0.47 *vs* 8.29 ± 0.41), the P value was not statistically significant. Patients with NSCLC were divided in to three groups according to Tumor-Node-Metastasis (TNM) stage: Tis (n = 130), TNM I (n = 395) and TNM II–IV (n = 266). Although the TyG index was slightly elevated in patients with TNM II–IV compared with patients with TNM I and Tis stage (8.44 ± 0.54 *vs* 8.42 ± 0.53 *vs* 8.41 ± 0.64), the TyG index was not statistically different among the three groups.

### The Value of the TyG Index for Predicting the Incident of NSCLC

**Figure 2:**
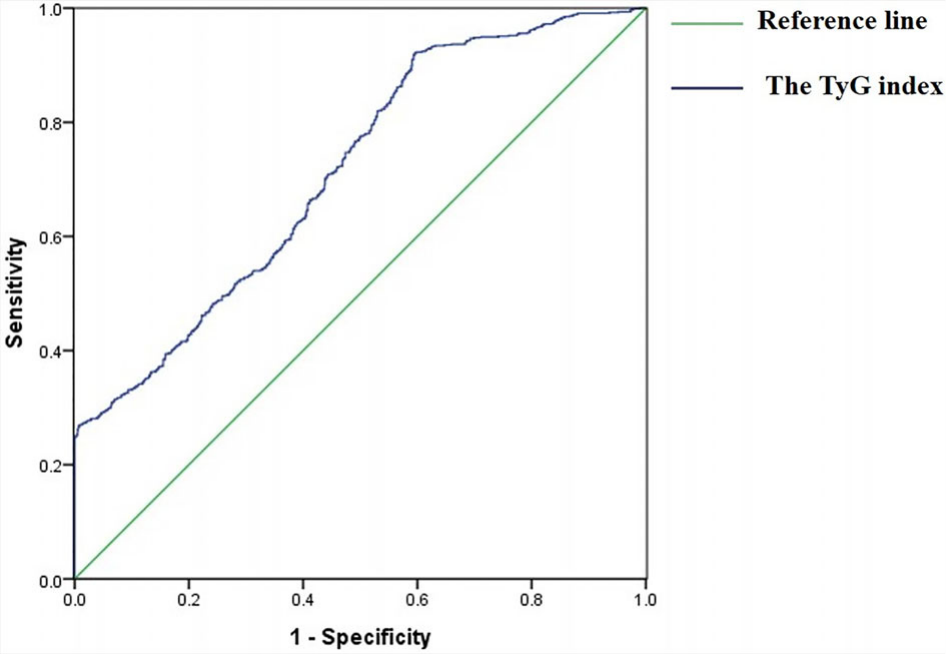
Receiver operative characteristic (ROC) curves of the TyG index for predicting the incident of NSCLC.

ROC analysis showed that the AUROC of the TyG index for predicting the incident of NSCLC was 0.713 (95%CI 0.688–0.738, *P* < 0.001), the optimal cut-off point for the TyG index was 8.18 (sensitivity: 70.9%, specificity: 60.2%), which indicated that the TyG index was an acceptable predictor for the incident of NSCLC (**Figure 2**).

## Discussion

In this study, an independent relationship between TyG index and NSCLC was observed after adjustment for conventional risk factors. To our knowledge, this is the first study to demonstrate this relationship between the TyG index and NSCLC. Furthermore, the results indicate that the TyG index may be an effective sign of NSCLC events.

Lung cancer is the leading cause of cancer-related death both in developed and developing countries. In China, the incidence of lung cancer in men and women has increased rapidly in recent years, causing a huge social and economic burden (2). Non-small cell lung cancer (NSCLC) is the most common subtype of lung cancer; however, the exact mechanism leading to NSCLC remains unclear. Several researchers have specifically studied the risk factors of lung cancer, including smoking, environmental pollution, chronic obstructive pulmonary disease, obesity, and dyslipidemia, and took corresponding measures to intervene (17–19). However, the incidence of lung cancer in China is still growing rapidly. Therefore, it is necessary to explore other NSCLC risk factors and improve prevention efficiency to reduce morbidity and mortality.

Insulin resistance (IR) is a condition with greater prevalence and significance in the context of obesity, Mets, NAFLD, and T2DM. In addition, emerging evidence suggested that insulin resistance was closely related to cancer incidence and mortality, including colorectal cancer (20), gastric cancer (21), and breast cancer (22). However, there was a considerable controversy about the association of IR with lung cancer. The U.S. National Health and Nutrition Examination Survey III reported that IR was more strongly associated with an increased risk of overall cancer mortality after the exclusion of lung cancer deaths (12), because lung cancer was generally considered to be independent of obesity. Argirion et al. found that higher fasting serum insulin concentrations, as well as the presence of IR were associated with an elevated risk of lung cancer development (11). IR may mediate cancer-related functions fairly distinct from its obesity-related aspects. Insulin resistance and elevated serum leptin levels were interrelated and jointly promoted lung cancerization (23). As we know, HOMA-IR has been a validated surrogate IR marker and widely used in clinical practice. However, since insulin measurement is not a routine examination for lung cancer patients, its clinical application is limited. The TyG index based on fasting triglycerides and glucose levels is another reliable and simple surrogate indicator of IR. In addition, some studies have shown that the predictive value of the TyG index for IR is better than HOMA-IR (24, 25). Recently, it has shown that TyG index was significantly associated with IR-related diseases and cardiovascular diseases (26, 27). A large prospective study involving 510,471 individuals from six European cohorts showed that the TyG index was associated with an increased risk of gastrointestinal tumors (28, 29). Although both triglycerides and glucose had been demonstrated to be associated with lung cancer risk (30, 31), few studies focused on the relationship between the TyG index and NSCLC. In this study, we found that the TyG index was significantly correlated with NSCLC risk even after adjusting for conventional risk factors. Meanwhile, the incidence of NSCLC increased with the increase of the TyG index. However, there were no differences of the TyG index in different histopathological types or TNM stages. ROC analysis showed that the AUROC of the TyG index for predicting NSCLC risk was 0.713, with a sensitivity of 70.9% and a specificity of 60.2%, which indicated that the TyG index may be a useful and reliable marker for NSCLC risk.

Although the mechanism underlying the relationship between the TyG index and NSCLC risk has not been fully elucidated, it may be related to IR. Higher circulating insulin levels are characteristic of IR and may potentially show cancer-promoting effects through various molecular mechanisms. On the one hand, insulin can promote the activity of insulin-like growth factor (IGF-I), which is an effective growth factor in promoting lung cancer growth (31). On the other hand, insulin is one of the main stimuli of the Ras signaling pathway which plays a key role in the onset of lung cancer (32). In addition, there were a few studies that showed that it may also promote tumor growth directly or indirectly through inflammatory pathways, leptin, and adiponectin (33, 34). The exact mechanism is still unclear and needs further clarification.

This study has several limitations. First of all, it is difficult to determine whether the TyG index has a causative effect on NSCLC because of the cross-sectional design. Secondly, the results indicate that after adjustment for inflammation makers including WBCC and neutrophil count, the correlation of the TyG index with NSCLC risk remained significant, but the potential role of TyG index in the risk of NSCLC caused by inflammation needs to be further studied because other inflammatory indicators such as C-reactive protein and TNF-α have not been detected and analyzed in the research. At last, information about passive smoking has not been available in this study.

## Conclusion

Our study firstly shows the evidence that the TyG index is independently associated with NSCLC risk. Thus, possible recommendations to encourage the general population to maintain normal TyG index may reduce the NSCLC risk. Certainly, multi-center and large-scale prospective studies are needed to confirm our results.

## Data Availability Statement

The original contributions presented in the study are included in the article/supplementary material. Further inquiries can be directed to the corresponding authors.

## Ethics Statement

The study was approved by the Human Research Ethics Committee of Nanjing Drum Tower Hospital.

## Author Contributions

XY: Writing—Original draft preparation. YG: Formal analysis, Validation, Investigation. JT: Data curation, Validation. MT: Data curation. JD: Project administration. YZ: Writing—Reviewing and editing, Supervision. All authors contributed to the article and approved the submitted version.

## Funding

This work was supported by the National Natural Science Foundation of China (Grant No. 81900062).

## Conflict of Interest

The authors declare that the research was conducted in the absence of any commercial or financial relationships that could be construed as a potential conflict of interest.

## References

[1] Bray F Ferlay J Soerjomataram I Siegel RL Torre LA Jemal A. Global cancer statistics 2018: GLOBOCAN estimates of incidence and mortality worldwide for 36 cancers in 185 countries. CA Cancer J Clin (2018) 68:394–424. 10.3322/caac.21492 30207593

[2] Yang D Liu Y Bai C Wang X Powell CA. Epidemiology of lung cancer and lung cancer screening programs in China and the United States. Cancer Lett (2020) 468:82–7. 10.1016/j.canlet.2019.10.009 31600530

[3] Barta JA Powell CA Wisnivesky JP. Global Epidemiology of Lung Cancer. Ann Glob Health (2019) 85(1):1–16. 10.5334/aogh.2419 30741509 PMC6724220

[4] Doukas SG Vageli DP Lazopoulos G Spandidos DA Sasaki CT Tsatsakis A. The Effect of NNK, A Tobacco Smoke Carcinogen, on the miRNA and Mismatch DNA Repair Expression Profiles in Lung and Head and Neck Squamous Cancer Cells. Cells (2020) 9(4):1031. 10.3390/cells9041031 PMC7226174 32326378

[5] Sasco AJ Secretan MB Straif K. Tobacco smoking and cancer: a brief review of recent epidemiological evidence. Lung Cancer (2004) 45 Suppl 2:S3–9. 10.1016/j.lungcan.2004.07.998 15552776

[6] Chen S Chen Y Liu X Li M Wu B Li Y. Insulin resistance and metabolic syndrome in normal-weight individuals. Endocrine (2014) 46:496–504. 10.1007/s12020-013-0079-8 24190050

[7] Fracanzani AL Valenti L Bugianesi E Andreoletti M Colli A Vanni E. Risk of severe liver disease in nonalcoholic fatty liver disease with normal aminotransferase levels: a role for insulin resistance and diabetes. Hepatology (2008) 48:792–8. 10.1002/hep.22429 18752331

[8] Lu CC Chu PY Hsia SM Wu CH Tung YT Yen GC. Insulin induction instigates cell proliferation and metastasis in human colorectal cancer cells. Int J Oncol (2017) 50:736–44. 10.3892/ijo.2017.3844 28101572

[9] Albanes D Weinstein SJ Wright ME Mannisto S Limburg PJ Snyder K. Serum insulin, glucose, indices of insulin resistance, and risk of prostate cancer. J Natl Cancer Inst (2009) 101:1272–9. 10.1093/jnci/djp260 PMC2744728 19700655

[10] Malaguarnera R Vella V Nicolosi ML Belfiore A. Insulin Resistance: Any Role in the Changing Epidemiology of Thyroid Cancer? Front Endocrinol (Lausanne) (2017) 8:314. 10.3389/fendo.2017.00314 29184536 PMC5694441

[11] Argirion I Weinstein SJ Mannisto S Albanes D Mondul AM. Serum Insulin, Glucose, Indices of Insulin Resistance, and Risk of Lung Cancer. Cancer Epidemiol Biomarkers Prev (2017) 26:1519–24. 10.1158/1055-9965.EPI-17-0293 PMC5626607 28698186

[12] Parekh N Lin Y Hayes RB Albu JB Lu-Yao GL. Longitudinal associations of blood markers of insulin and glucose metabolism and cancer mortality in the third National Health and Nutrition Examination Survey. Cancer Causes Control (2010) 21:631–42. 10.1007/s10552-009-9492-y PMC3817266 20094767

[13] Pahkala K Laitinen TT Niinikoski H Kartiosuo N Rovio SP Lagstrom H. Effects of 20-year infancy-onset dietary counselling on cardiometabolic risk factors in the Special Turku Coronary Risk Factor Intervention Project (STRIP): 6-year post-intervention follow-up. Lancet Child Adolesc Health (2020) 4:359–69. 10.1016/S2352-4642(20)30059-6 32333883

[14] Mohd Nor NS Lee S Bacha F Tfayli H Arslanian S. Triglyceride glucose index as a surrogate measure of insulin sensitivity in obese adolescents with normoglycemia, prediabetes, and type 2 diabetes mellitus: comparison with the hyperinsulinemic-euglycemic clamp. Pediatr Diabetes (2016) 17:458–65. 10.1111/pedi.12303 26251318

[15] Lee SB Kim MK Kang S Park K Kim JH Baik SJ. Triglyceride Glucose Index Is Superior to the Homeostasis Model Assessment of Insulin Resistance for Predicting Nonalcoholic Fatty Liver Disease in Korean Adults. Endocrinol Metab (Seoul) (2019) 34:179–86. 10.3803/EnM.2019.34.2.179 PMC6599902 31257745

[16] Lee SB Ahn CW Lee BK Kang S Nam JS You JH. Association between triglyceride glucose index and arterial stiffness in Korean adults. Cardiovasc Diabetol (2018) 17:41. 10.1186/s12933-018-0692-1 29562908 PMC5863385

[17] Andreotti G Freedman ND Silverman DT Lerro CC Koutros S Hartge P. Tobacco Use and Cancer Risk in the Agricultural Health Study. Cancer Epidemiol Biomarkers Prev (2017) 26:769–78. 10.1158/1055-9965.EPI-16-0748 PMC5413369 28035020

[18] Lyu Z Li N Wang G Feng X Chen S Su K. Independent and joint associations of blood lipids and lipoproteins with lung cancer risk in Chinese males: A prospective cohort study. Int J Cancer (2019) 144:2972–84. 10.1002/ijc.32051 30536993

[19] Zhang X Liu Y Shao H Zheng X. Obesity Paradox in Lung Cancer Prognosis: Evolving Biological Insights and Clinical Implications. J Thorac Oncol (2017) 12:1478–88. 10.1016/j.jtho.2017.07.022 28757418

[20] Obi K Ramsey M Hinton A Stanich P Gray DM,2 Krishna SG. Insights into insulin resistance, lifestyle, and anthropometric measures of patients with prior colorectal cancer compared to controls: A National Health and Nutrition Examination Survey (NHANES) Study. Curr Probl Cancer (2018) 42:276–85. 10.1016/j.currproblcancer.2017.12.002 29395416

[21] Kwon HJ Park MI Park SJ Moon W Kim SE Kim JH. Insulin Resistance Is Associated with Early Gastric Cancer: A Prospective Multicenter Case Control Study. Gut Liver (2019) 13:154–60. 10.5009/gnl17556 PMC6430436 30400721

[22] Jung SY Papp JC Sobel EM Yu H Zhang ZF. Breast Cancer Risk and Insulin Resistance: Post Genome-Wide Gene-Environment Interaction Study Using a Random Survival Forest. Cancer Res (2019) 79:2784–94. 10.1158/0008-5472.CAN-18-3688 PMC6522308 30936085

[23] Petridou ET Sergentanis TN Antonopoulos CN Dessypris N Matsoukis IL Aronis K. Insulin resistance: an independent risk factor for lung cancer? Metabolism (2011) 60:1100–6. 10.1016/j.metabol.2010.12.002 21251684

[24] Vasques AC Novaes FS de Oliveira Mda S Souza JR Yamanaka A Pareja JC. TyG index performs better than HOMA in a Brazilian population: a hyperglycemic clamp validated study. Diabetes Res Clin Pract (2011) 93:e98–e100. 10.1016/j.diabres.2011.05.030 21665314

[25] Lee SH Kwon HS Park YM Ha HS Jeong SH Yang HK. Predicting the development of diabetes using the product of triglycerides and glucose: the Chungju Metabolic Disease Cohort (CMC) study. PloS One (2014) 9:e90430. 10.1371/journal.pone.0090430 24587359 PMC3938726

[26] Zheng R Du Z Wang M Mao Y Mao W. A longitudinal epidemiological study on the triglyceride and glucose index and the incident nonalcoholic fatty liver disease. Lipids Health Dis (2018) 17:262. 10.1186/s12944-018-0913-3 30458848 PMC6247753

[27] Kim MK Ahn CW Kang S Nam JS Kim KR Park JS. Relationship between the triglyceride glucose index and coronary artery calcification in Korean adults. Cardiovasc Diabetol (2017) 16:108. 10.1186/s12933-017-0589-4 28830471 PMC5568209

[28] Fritz J Bjorge T Nagel G Manjer J Engeland A Haggstrom C. The triglyceride-glucose index as a measure of insulin resistance and risk of obesity-related cancers. Int J Epidemiol (2020) 49:193–204. 10.1093/ije/dyz053 30945727

[29] Zuber V Marconett CN Shi J Hua X Wheeler W Yang C. Pleiotropic Analysis of Lung Cancer and Blood Triglycerides. J Natl Cancer Inst (2016) 108(12):djw167. 10.1093/jnci/djw167 27565901 PMC5241892

[30] Bergamino M Rullan AJ Saigi M Peiro I Montanya E Palmero R. Fasting plasma glucose is an independent predictor of survival in patients with locally advanced non-small cell lung cancer treated with concurrent chemoradiotherapy. BMC Cancer (2019) 19:165. 10.1186/s12885-019-5370-5 30791870 PMC6385407

[31] Kim WY Jin Q Oh SH Kim ES Yang YJ Lee DH. Elevated epithelial insulin-like growth factor expression is a risk factor for lung cancer development. Cancer Res (2009) 69:7439–48. 10.1158/0008-5472.CAN-08-3792 PMC2745504 19738076

[32] Mascaux C Iannino N Martin B Paesmans M Berghmans T Dusart M. The role of RAS oncogene in survival of patients with lung cancer: a systematic review of the literature with meta-analysis. Br J Cancer (2005) 92:131–9. 10.1038/sj.bjc.6602258 PMC2361730 15597105

[33] Wang F Zhang L Sai B Wang L Zhang X Zheng L. BMSC-derived leptin and IGFBP2 promote erlotinib resistance in lung adenocarcinoma cells through IGF-1R activation in hypoxic environment. Cancer Biol Ther (2020) 21:61–71. 10.1080/15384047.2019.1665952 31559898 PMC7012080

[34] Li F Cao Y Li J Gao C Dong X Ren P. The clinical significance of serum adipocytokines level in patients with lung cancer. J Thorac Dis (2019) 11:3547–55. 10.21037/jtd.2019.07.66 PMC6753436 31559061

